# Predicting vasospasm risk using first presentation aneurysmal subarachnoid hemorrhage volume: A semi-automated CT image segmentation analysis using ITK-SNAP

**DOI:** 10.1371/journal.pone.0286485

**Published:** 2023-06-01

**Authors:** James S. Street, Anand S. Pandit, Ahmed K. Toma

**Affiliations:** 1 Department of Clinical and Experimental Epilepsy, Institute of Neurology, University College London, London, United Kingdom; 2 Victor Horsley Department of Neurosurgery, The National Hospital for Neurology and Neurosurgery, Queen Square, London, United Kingdom; 3 High-Dimensional Neurology, Institute of Neurology, University College London, London, United Kingdom; Universitatsklinikum Regensburg, GERMANY

## Abstract

**Purpose:**

Cerebral vasospasm following aneurysmal subarachnoid hemorrhage (aSAH) is a significant complication associated with poor neurological outcomes. We present a novel, semi-automated pipeline, implemented in the open-source medical imaging analysis software ITK-SNAP, to segment subarachnoid blood volume from initial CT head (CTH) scans and use this to predict future radiological vasospasm.

**Methods:**

42 patients were admitted between February 2020 and December 2021 to our tertiary neurosciences center, and whose initial referral CTH scan was used for this retrospective cohort study. Blood load was segmented using a semi-automated random forest classifier and active contour evolution implemented in ITK-SNAP. Clinical data were extracted from electronic healthcare records in order to fit models aimed at predicting radiological vasospasm risk.

**Results:**

Semi-automated segmentations demonstrated excellent agreement with manual, expert-derived volumes (mean Dice coefficient = 0.92). Total normalized blood volume, extracted from CTH images at first presentation, was significantly associated with greater odds of later radiological vasospasm, increasing by approximately 7% for each additional cm^3^ of blood (OR = 1.069, 95% CI: 1.021–1.120; p < .005). Greater blood volume was also significantly associated with vasospasm of a higher Lindegaard ratio, of longer duration, and a greater number of discrete episodes. Total blood volume predicted radiological vasospasm with a greater accuracy as compared to the modified Fisher scale (AUC = 0.86 vs 0.70), and was of independent predictive value.

**Conclusion:**

Semi-automated methods provide a plausible pipeline for the segmentation of blood from CT head images in aSAH, and total blood volume is a robust, extendable predictor of radiological vasospasm, outperforming the modified Fisher scale. Greater subarachnoid blood volume significantly increases the odds of subsequent vasospasm, its time course and its severity.

## Introduction

Outcomes following aneurysmal subarachnoid hemorrhage (aSAH) remain poor, with an estimated mortality of approximately 30% [1]. An important contributor to both morbidity and mortality following aSAH is cerebral vasospasm, the spasmodic narrowing of intracranial arteries which can lead to delayed cerebral ischaemia (DCI). Symptomatic vasospasm occurs in 20% of patients [2], and typically occurs at a delay of 3 to 12 days after the haemorrhagic event [3]. Whereas angiographic or radiologically-detected vasospasm can be detected in as many as 50–70% of aSAH patients, not all are associated with neurological deficits. As such, the prediction and forward recognition of clinically significant vasospasm represents a substantial challenge in the management of these patients.

One predictor of future vasospasm is the total volume of blood seen on the CT head scan at presentation [4–8]. Furthermore, intraventricular hemorrhage (IVH) has been shown to independently predict cerebral vasospasm [6, 8–13], and total blood volume is associated with worse functional outcomes [14]. Drawing on these key findings, aSAH severity is frequently graded in clinical practice using the modified Fisher scale (mFS) [8]—a subjective assessment of bleed extent on CT head scans. However, the modified Fisher scale only crudely notes blood distribution and blood load, and accordingly, its qualitative nature limits its predictive power. Troublingly, the modified Fisher scale has recently been demonstrated to lack inter-rater reliability [15], and thus may not provide an objective metric of blood burden and distribution following aSAH.

Further work has introduced similar qualitative or semi-quantitative severity scores for the purposes of predicting vasospasm from cisternal blood volume [5], intraventricular blood volume [16], and intraparenchymal blood volume [17]. Yet, these scales are also observer-dependent and can exhibit poor inter-rater agreement [18, 19]. Further, although the presence and degree of blood in cerebral compartments is associated with clinical, symptomatic vasospasm [8, 12, 19], little work has looked at the prediction of radiological vasospasm from routinely acquired neuroimaging data. Although the precise relationship between radiological vasospasm and DCI is contested [20], there is strong recent evidence of its association with clinical and functional outcomes [21, 22]. As the onset of DCI is difficult to diagnose and frequently missed, especially in patients of poor clinical grade whose neurology is difficult to assess [23], such radiological outcomes remain important to guide decisions regarding clinical intervention, including angioplasty [24].

Quantitative image segmentation approaches, such as those used to segment other organ systems [25], can detail both the volume and morphology of blood load and would overcome the aforementioned difficulties, potentially offering a more accurate method for vasospasm prediction. Novel developments in medical image segmentation mean that precise and robust estimates of blood volume and distribution are easier to calculate. These include semi-automated tools such as active contour evolution and clustering based algorithms [26] which are more efficient and have precedent in delineating vascular structures in other areas [27].

Here, we present a working pipeline, implemented in ITK-SNAP, for the efficient, semi-automated segmentation of blood on a non-contrast, routine plain CT head scan, obtained at first presentation of patients with aSAH. We demonstrate that semi-automated segmentations align well with clinical expert impressions of blood distribution and require minimal correction. Our model is compared against the current standard of the modified Fisher scale in correlating against occurrence of any radiological vasospasm as the primary outcome. Secondary outcomes included time to, duration and number of discrete vasospasm episodes and general reported outcome measures of length of intensive therapy unit (ITU) stay, hospital stay and mortality.

## Methods

### Protocol

The study was performed in accordance with the STROBE checklist [28] and the European Society of Radiology statement on imaging biomarkers [29] where relevant.

### Ethics

This retrospective cohort study was approved by the institutional review board (53-202122-CA) in the context of a wider service evaluation regarding radiological assessment of vasospasm in patients with aSAH at our tertiary neurosciences center. The need for consent for anonymised data usage was waived whereas all patients gave their written consent for any interventional procedures.

### Participants

A list of candidate patients were identified from the institutional electronic healthcare record (EHR) Patients were included in this study if they (i) were treated for an aneurysmal subarachnoid hemorrhage at the academic neurosciences center between February 2020—December 2021; (ii) the initial CT head (CTH) scan performed at first presentation was available on the clinical imaging repository; and (iii) the patient had no prior medical history of aSAH or intracranial hemorrhage, no previous intracranial coil embolisation, or any other intracranial implant in situ that would degrade CT image quality.

### Image processing and segmentation pipeline

**Fig 1:**
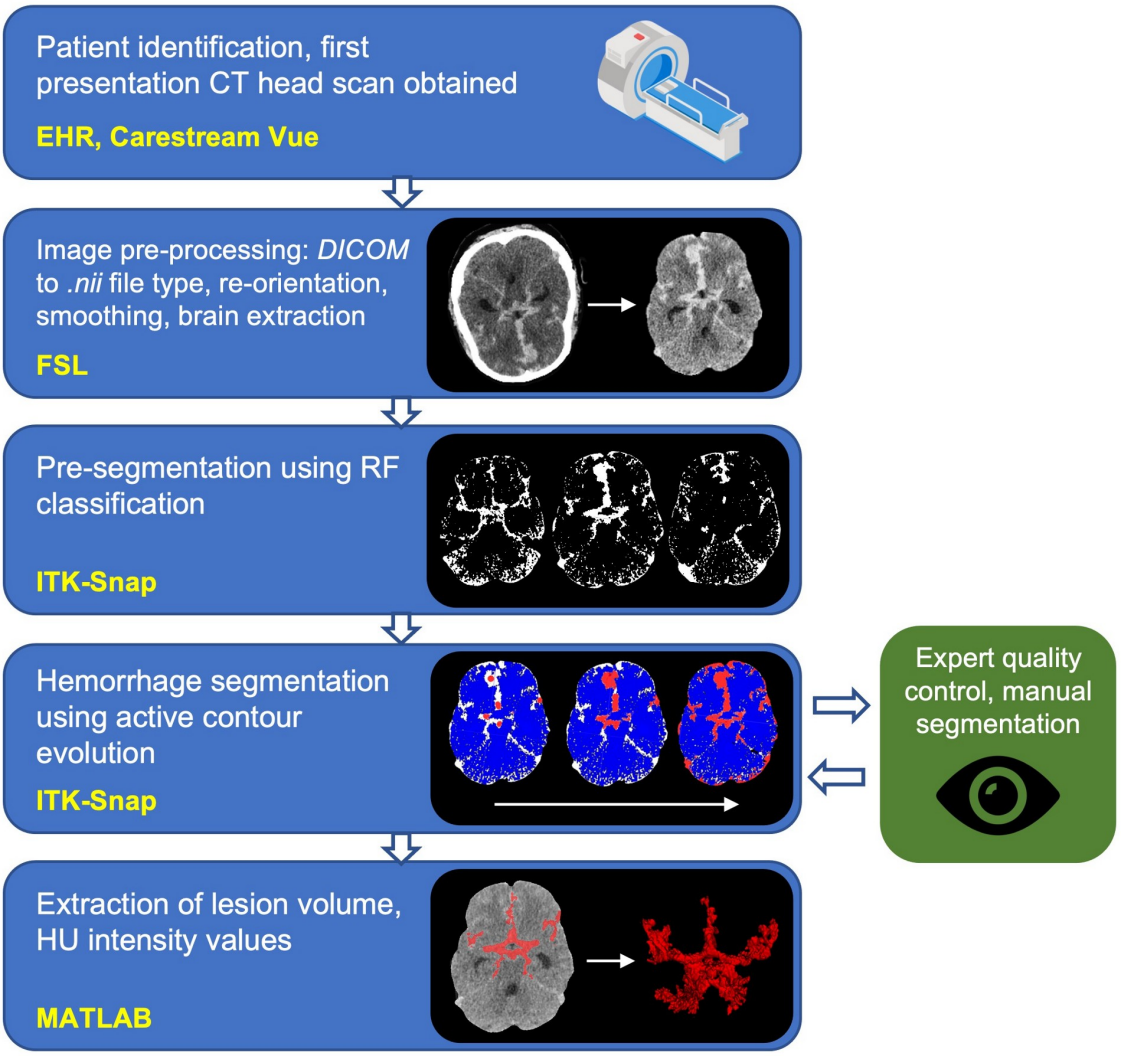
Image processing pipeline describing data collection, pre-processing, segmentation, quality control and information extraction. (yellow font = software library; DICOM = Digital Imaging and Communications in Medicine; nii = nifti file type; FSL = FMRIB Software Library; ITK-Snap = Insight Segmentation and Registration Toolkit; RF = random forest; HU = Hounsfield Units. ‘CT-scan’ designed using resources from Flatiron.com).

The steps regarding image pre- and post-processing and hemorrhage segmentation, alongside the software packages used have been outlined in Fig 1. Briefly, the first CT head (CTH) scan performed following the SAH ictus was obtained from the Picture Archiving and Communication System (PACS). DICOM CT files were anonymised and converted to NIfTI format using the command line tool *dcm2niix* [30]. *dcm2niix* includes an inbuilt Gantry tilt correction routine that was used to ensure NIfTI files were correctly oriented. NIfTI files were loaded into the FMRIB software library (FSL) [31] and a binary mask image was generated to delineate the brain using FSL’s Brain Extraction Tool (BET). To perform BET, images were initially smoothed using a Gaussian kernel of size 1mm^3^ and thresholded between 0 to 120 Hounsfield Units (HU) [32] and parameterised for optimal extraction of brain tissue from CT images [32]. The extracted brain image was binarised to generate a brain mask that could be used in subsequent analysis, and was manually inspected for adequacy before being used.

**Fig 2:**
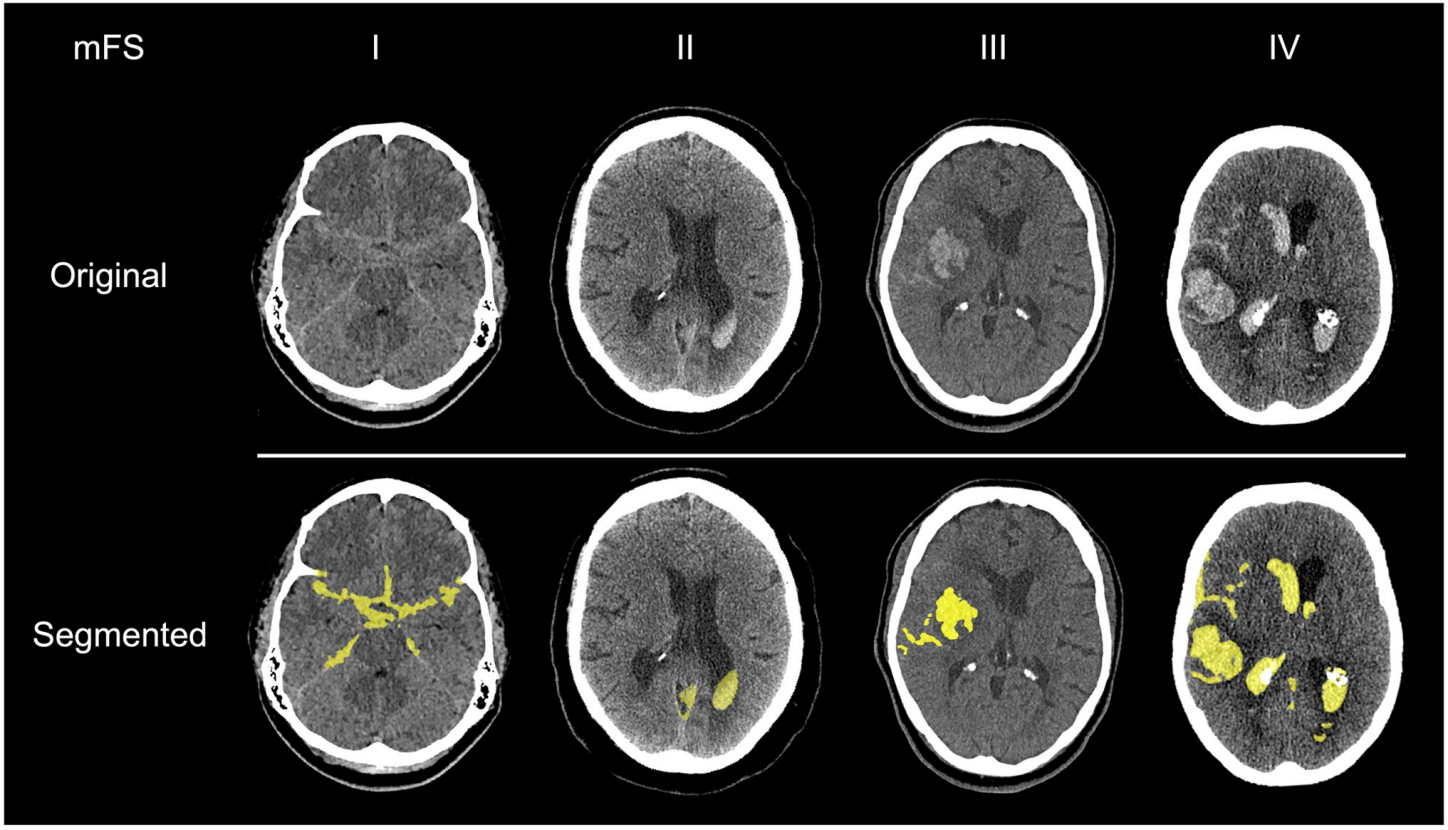
Examples of semi-automated subarachnoid hemorrhage segmentations for each modified Fisher grade. (yellow = segmentation overlay, mFS = modified Fisher Scale).

All segmentations were performed in ITK-SNAP, an open-source and multi-platform 3D medical image analysis software, optimized for user-guided segmentation [33]. All images were resampled using linear interpolation such that voxels were cubic in size to ensure consistency between patients and diverse scanner types. Briefly, to segment the image, a random forest classifier (tree depth = 30, number of trees = 50, classifier bias = 0.5) was initially trained on examples within each image, which were labeled by the experimenter (J.S.S.) for each scan, to classify voxels into one of four tissue subtypes: CSF, bone, parenchyma, or blood (Fig 1). HU values at each labeled voxel and the 2-voxel wide neighborhood around each labeled voxel were used to train the classifier. This was used to generate a speed image, which encoded at each pixel the desired rate of growth or retraction of the contour. Spherical seeds were manually placed on the image to initialize the contour, which was then allowed to actively evolve over approximately 500 iterations. Segmentations took, on average, 12 minutes to perform for each brain. Example series of axial slices through four brains are shown in Fig 2, which display the segmentation output for all blood detected by this process.

### Quality assessment

All segmentations were reviewed and manually corrected by an academic neurosurgical resident with a decade of post-doctoral neuroimaging experience (A.S.P.) and consultant neurovascular surgeon (A.K.T.) to provide finalized segmentations to act as the ground truth for subsequent analyses. We ensured that the experimenter who labeled the training data (J.S.S.) was different from the experimenters who provided expert corrections (A.S.P. and A.K.T.) so as to minimize the introduction of bias into the training data provided to the model. Dice overlap coefficients were calculated between the original and expert-corrected segmentations to assess the quality of the semi-automated segmentation pipeline.

### Clinical data extraction

Relevant clinical data were extracted from the EHR and from radiology reports available on PACS. This data included, as the primary outcome, the presence of radiologically detectable vasospasm via: transcranial doppler (TCD) ultrasound imaging of the major intracranial vessels, CT angiogram (CTA), or digital subtraction angiography (DSA). The majority of patients received repeat TCD imaging during recovery, with documented Lindegaard ratios (n = 30), with CTA and DSA performed based on assessment of clinical need. Secondary outcomes included time to vasospasm, vasospasm duration (defined as the number of days with any positive radiological vasospasm), number of discrete vasospasm episodes (defined as the number of detected vasospasm that were separated by at least one day of exclusively negative tests), vasospasm severity, length of ITU stay, length of hospital stay, and mortality. Vasospasm severity was quantified in two ways. Firstly, the consultant neuroradiologist’s subjective impression of vessel caliber (using CTA or DSA) was extracted and categorized as: ‘none’, ‘mild’, ‘moderate’, or ‘severe’. Secondly, the greatest Lindegaard ratio was extracted from radiology records for all patients who received TCD imaging and where this was documented. Given that many of the patients were intubated and sedated during their initial hospital admission and the data was retrospectively collected, clinical vasospasm or delayed clinical ischaemia (DCI) could not be reliably ascertained. The modified Fisher grade was recorded by author A.S.P following manual review of the images.

### Data and statistical analysis

Generalized linear models were fitted in MATLAB (R2019b, Mathworks Inc., Natick, MA) to assess for significant associations between predictors and response variables. Where response variables were binary values, logistic regression was used, otherwise a linear regression model was used.

For logistic models, (McFadden’s) pseudo-R^2^, was calculated to estimate the model’s predictive power [34], with values between 0.2–0.4 representing an “excellent” model fit [35]. Model goodness-of-fit was calculated in reference to a null model fit with constant (intercept) terms alone. To compare model performance, we used previously described methods to estimate a z-score for the difference in each model’s area under the curve (AUC) [36]. Additional comparisons between nested models were performed using the likelihood-ratio test, alongside evaluation of the Akaike information criterion (AIC) and Bayesian information criterion (BIC). Hosmer-Lemeshow and Stukel tests were used to assess for model fitting and misspecification (Supplementary Methods in S1 File).

All values are reported as mean ± standard deviation, unless otherwise specified. Data were tested for normality using the one-sample Kolmogorov-Smirnov test. All significance tests, unless stated otherwise, were two-tailed with a significance threshold of 5%. Total blood volume was normalized and scaled *(total blood volume x mean participant brain volume / participant brain volume)* using the brain volume estimated from the BET images derived above. Blood volume reported is normalized to participant brain volume unless stated otherwise. The sample size was determined pragmatically, namely the maximum number of images obtained within the specified study period. Scatter plots, unless stated otherwise, are color-coded according to the radiological vasospasm status of the patient (maroon: vasospasm detected by any modality during hospital stay; blue: no vasospasm detected by any modality during hospital stay).

## Results

### Participant demographics

Data from 42 patients were used for this study (see Table 1 for a summary of demographics). 71.4% (n = 30) of the included patients developed radiological vasospasm, detected via TCD, CTA, or DSA during their hospital stay, comparable to previously reported rates of radiological vasospasm [3]. There was no evidence of a difference in vasospasm risk in patients following endovascular coil embolisation versus aneurysm clipping (χ^2^ = 1.45; p = 0.23), nor was vasospasm risk associated with patient gender (χ^2^ = 26; p = 0.61) or age (t = -1.00, p = 0.32). Patients with radiological vasospasm experienced similar ITU stays (360±202 hours vs 288±381 hours; p = 0.46), but remained in hospital for significantly longer as compared to those who did not (59.8±46.6 days vs 23.7±24.2 days; 95% CI [4.6, 67.6], t = 2.33, p = 0.026). Length of ITU stay was neither significantly correlated with the duration (r = 0.25; p = 0.14) or number of distinct vasospasm episodes (r = 0.09; p = 0.56).

**Table 1:** Descriptive information regarding each patient’s demographics, aneurysm, treatment and hospital outcomes.

**Number of patients**	42	
**Mean age (SD)**	57.8 (11.6) years	
**Female sex (%)**	27 (64.3)	
**Aneurysmal location (%)**	AComm	17 (40.5)
	PComm	5 (11.9)
	MCA	7 (16.7)
	ICA	5 (11.9)
	PICA	6 (14.3)
	SCA	2 (4.8)
**Radiological vasospasm frequency determined by (%)**	TCD	23 (54.8)
	CTA	30 (71.4)
	DSA	16 (38.1)
	Any modality	30 (71.4)
**Treatment frequency: CSF diversion (%)**	External ventricular drain	30 (71.4)
	Lumbar drain	11 (26.2)
	No CSF diversion	11 (26.2)
**Treatment frequency: Neurovascular (%)**	Coil embolisation	32 (76.2)
	Aneurysmal clipping	9 (21.4)
	*Other	1 (2.4)
**Mean length of ITU stay (SD)**	343 (251) hours	
**Mean hospital stay (SD)**	50.3 (44.6) days	
**Mortality (%)**	4 (9.5)	

(*patient passed away before coil embolisation organized. AComm = anterior communicating artery, PComm = posterior communicating artery, MCA = middle cerebral artery, ICA = internal carotid artery, PICA = posterior inferior cerebellar artery, SCA = superior cerebellar artery).

### Subarachnoid blood volume can be accurately and reliably segmented and estimated using a semi-automated pipeline

In general, all segmentations agreed with the expert-corrected segmentations (Dice coefficient: median = 0.994; mean = 0.920) with a mean volumetric error of (mean = 2.49±5.82 cm^3^).

### Segmented subarachnoid blood volume is associated with radiological vasospasm risk

**Fig 3:**
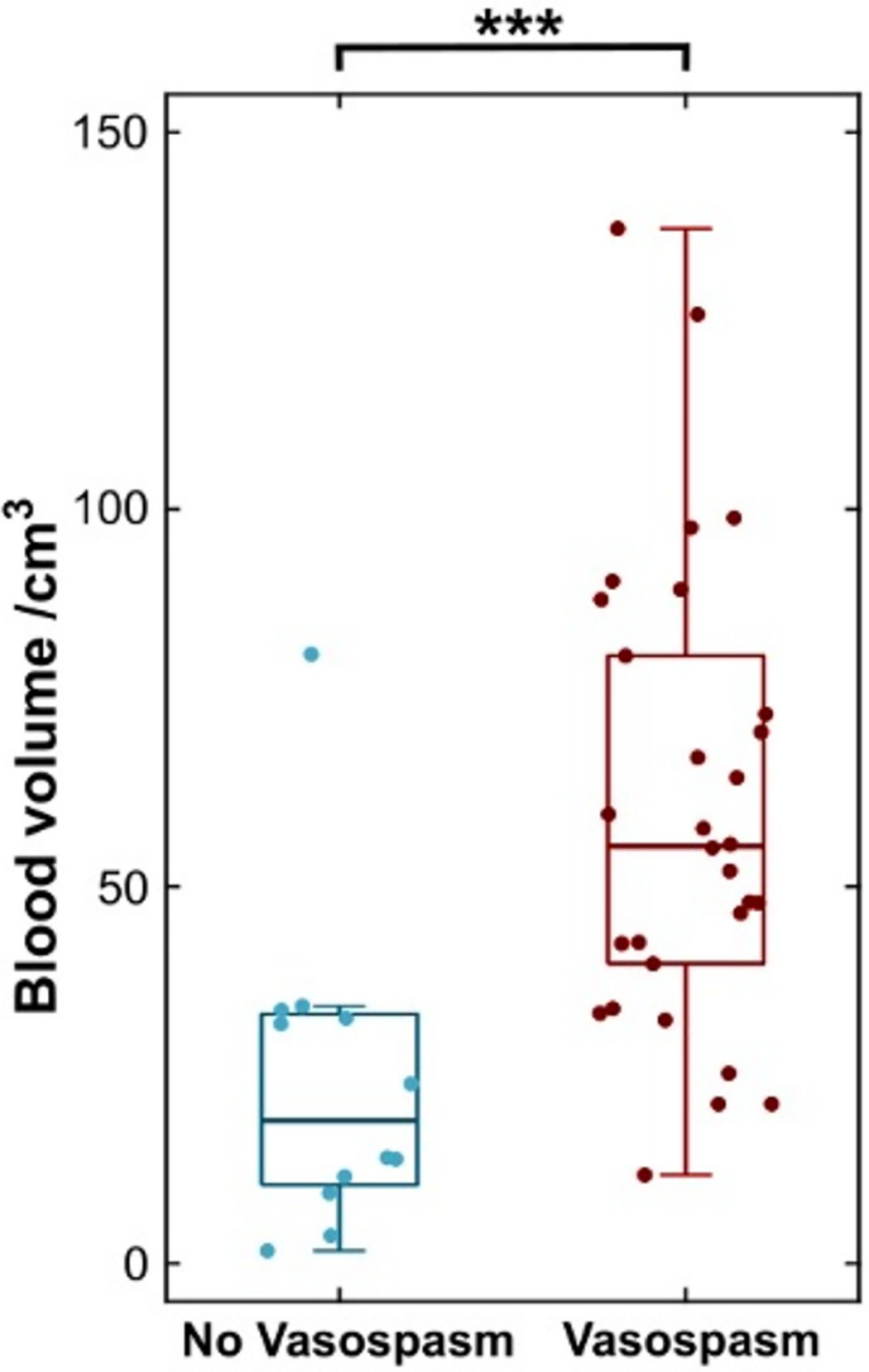
Greater segmented blood load is associated with greater radiological vasospasm risk. Boxplot of blood volume in patients who developed radiological vasospasm (maroon) and those who did not (blue). (*** = p< .001).

Patients who developed radiological vasospasm had a significantly greater non-normalised blood load on the CT head scan at initial presentation (vasospasm mean blood volume = 60.3±30.5 cm^3^; non-vasospasm mean blood volume = 24.2±21.4 cm^3^, t = 3.74, p < .001; Fig 3).

**Fig 4:**
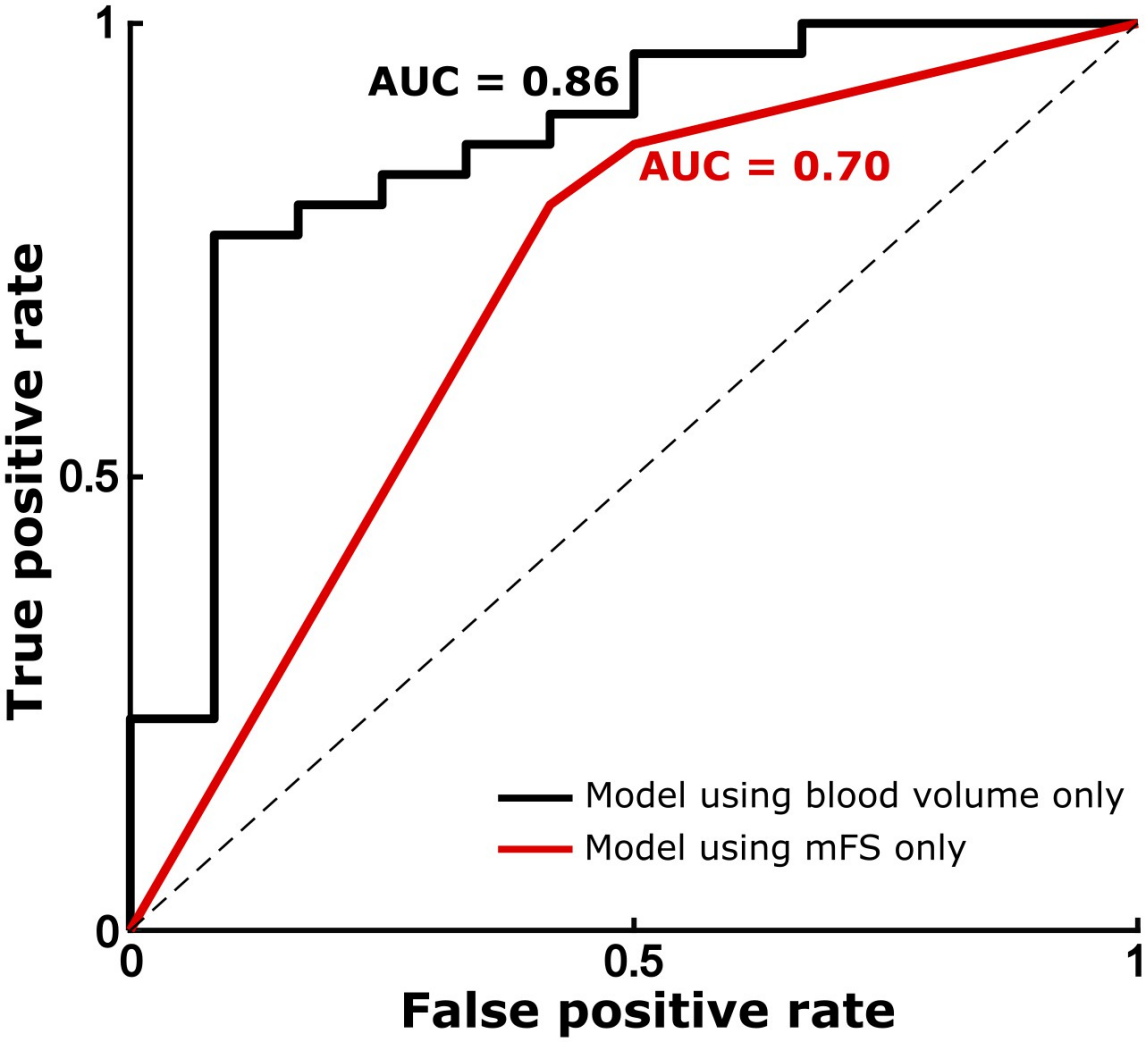
Receiver operating characteristic (ROC) curve demonstrating the performance characteristics of the binary classifier fit in the logistic regression model. Black = univariate logistic regression model using normalized total blood volume; gray = logistic regression model using dummy-coded modified Fisher score values.

Similarly, logistic regression demonstrated a significant association between normalized blood volume and vasospasm risk (OR = 1.069 [95% CI: 1.021–1.120]; p = .0049; df = 40). This indicates that the odds of radiological vasospasm occurring following admission increase by approximately 7% for each cm^3^ of blood present in the subarachnoid spaces. Based on our cohort, this model predicted that the threshold (for above 50% risk) for radiological vasospasm would occur at approximately 24.9cm^3^ of blood load, with 75.5cm^3^. This model explained a reasonable proportion of variance (pseudo-R^2^ = 0.30) and significantly more variance than a constant model (F = 15.2; p < .0001). There was no evidence that the model was misspecified (Stukel test: p_za_ = 0.61, p_zb_ = 0.51; Hosmer-Lemeshow test: χ^2^ = 3.49, df = 8, p = 0.90). A retriever operator characteristic (ROC) curve was constructed for this model (Fig 4). This demonstrated that the fitted model reliably separated the binary classes using normalized blood volume alone, and the ROC curve accordingly shows a high area-under-the-curve (AUC = 0.86). Furthermore, the significance of the relationship between blood volume and vasospasm was preserved after adding potential confounding variables into the model (see Table 2). Leave-one-out cross validation over this full model demonstrated a classification accuracy of 71.4% for subsequent vasospasm, and precision, sensitivity, and F1 of 80.0%. The full model maintained a high predictive power (AUC = 0.89) and good proportion of explained variance (pseudo-R^2^ = 0.41).

**Table 2:** Logistic regression fit parameters for all variables in the full predictive model including confounding variables.

	Estimate (log odds)	Confidence interval	*t* value	*p* value
**Intercept**	2.3975	[-4.40, 9.20]	0.69	0.49
**Age**	-0.099725	[-0.22, 0.02]	-1.69	0.09
**Gender**	1.4887	[-0.57, 3.54]	1.42	0.16
**Treatment: coiled**	0.14742	[-2.03, 2.33]	0.13	0.89
**EVD inserted**	0.78481	[-1.43, 3.00]	0.70	0.49
**Lumbar drain inserted**	0.08629	[-2.26 2.43]	0.072	0.94
Normalized blood volume*	*0*.*0741*	*[0*.*0077*, *0*.*1406]*	*2*.*19*	*0*.*03*

Following inclusion of potential confound variables into the logistic regression against radiological vasospasm, the only significant predictor remained the estimate for blood volume (*, p < .05, italic typeface). Note that estimates for logistic regression are given in the form of log odds. χ^2^-statistic vs. constant model: F = 20.6, p-value = 0.00215.

For comparison, a logistic regression model using only mFS scores was also fitted (see Supplementary Results S1 Table in S1 File). For dummy coding, mFS scores 1 and 2 were combined. While this also outperformed a constant model (χ^2^ = 6.31; p < .043), it had less predictive ability (AUC = 0.70) as compared to the previous models described (Fig 4). When directly comparing the two models [36], we found that the AUC of the univariate model using normalized blood volume was significantly greater than that using modified Fisher score (z = 1.86, p = 0.031), indicating that normalized blood volume model significantly outperformed the mFS model at predicting radiological vasospasm.

Greater scores on the mFS were significantly associated with larger volumes of subarachnoid blood (normalized blood volume for mFS 1: 8.5±5.7 cm^3^; mFS 2: 23.6±12.8 cm^3^; mFS 3: 53.9±37.8 cm^3^; mFS 4: 61.3±29.0 cm^3^; one-way ANOVA: F = 7.35, p < .001). Following Bonferroni correction for multiple comparisons, scans of mFS grade 4 contained significantly larger blood volume than scans of mFS grade 1 (p = .001) and grade 2 (p = .037). To assess whether blood volume contained additional independent information, it was added to a logistic regression model containing mFS alongside confounding variables (see Table 2, S2, S3 Tables in S1 File for full models). In doing so, model predictive power was improved (Likelihood ratio = 8.00; p < .0047) and predictive error was reduced (mFS only: AIC = 47.5, BIC = 58.0; mFS and blood volume: AIC = 41.55, BIC = 53.7). However, when the mFS was added to the blood volume model, no increase in predictive power was shown (Likelihood ratio = 1.94; p = 0.16). Similarly, leave-one-out cross validation of the full model demonstrated a classification accuracy of 78.6%, greater than the classification error for models containing blood volume (71.4%) or mFS (69.0%) alone. Taken together, these results indicate that the normalized blood volume contains additional information to the mFS, and constitutes a significant and independent predictor of radiological vasospasm.

### Greater subarachnoid blood volume is associated with worse vasospasm severity

**Fig 5:**
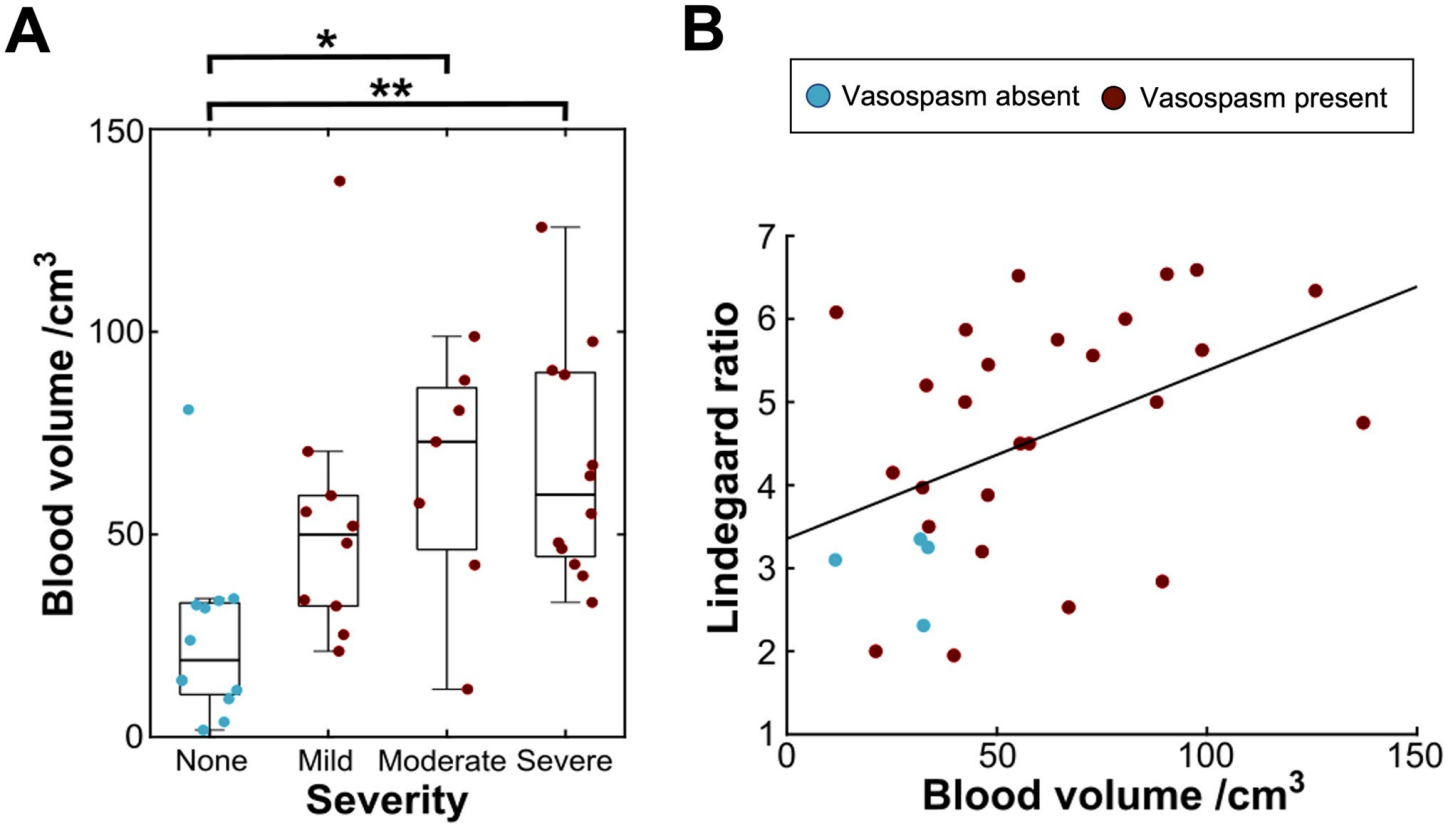
Associations between subarachnoid blood volume and metrics of severity of radiological vasospasm. A: Boxplots of blood volume grouped by radiologist’s impression of subjective severity of vasospasm. B: Scatter plot showing the greatest recorded Lindegaard ratio from TCD plotted against normalized blood volume. (* = p < .05, ** = p < .01).

To investigate whether segmented blood load was associated with the severity of radiological vasospasm, we extracted the subjective impression of severity from radiologist reports. A one-way ANOVA demonstrated a significant association between normalized blood volume and subjective severity (F = 5.42; p = 0.003). Post-hoc testing using the Tukey-Kramer method for multiple comparisons demonstrated that patients with reported ‘moderate’ and ‘severe’ radiological vasospasm had significantly greater blood load than those without vasospasm (p = 0.028 and p = 0.004, respectively; Fig 5A). However, no significant difference in blood volume was found between patients with mild, moderate, and severe vasospasm (p > .05 throughout).

To further probe this result, we evaluated quantitative metrics of vasospasm severity (Fig 5B). We extracted the largest Lindegaard ratio observed for all patients who received documented TCDs (n = 30 patients received at least one TCD positive or negative for vasospasm following admission). Similarly, blood volume was significantly correlated with higher Lindegaard ratios (r = 0.45; p = 0.014), indicating that blood load was related to the severity of ultrasonographic changes seen in vasospasm-positive patients. This relationship remained significant when only TCD-positive vasospasm patients were included (n = 23; r = 0.46; p = 0.027).

### Subarachnoid blood volume influences the duration and frequency of vasospasm episodes

Of patients who developed vasospasm, radiological evidence of vasospasm was first reported an average of 5.30±3.41 days after presentation, and persisted for 3.62±3.22 days, consisting of 1.29±1.22 discrete episodes.

**Fig 6:**
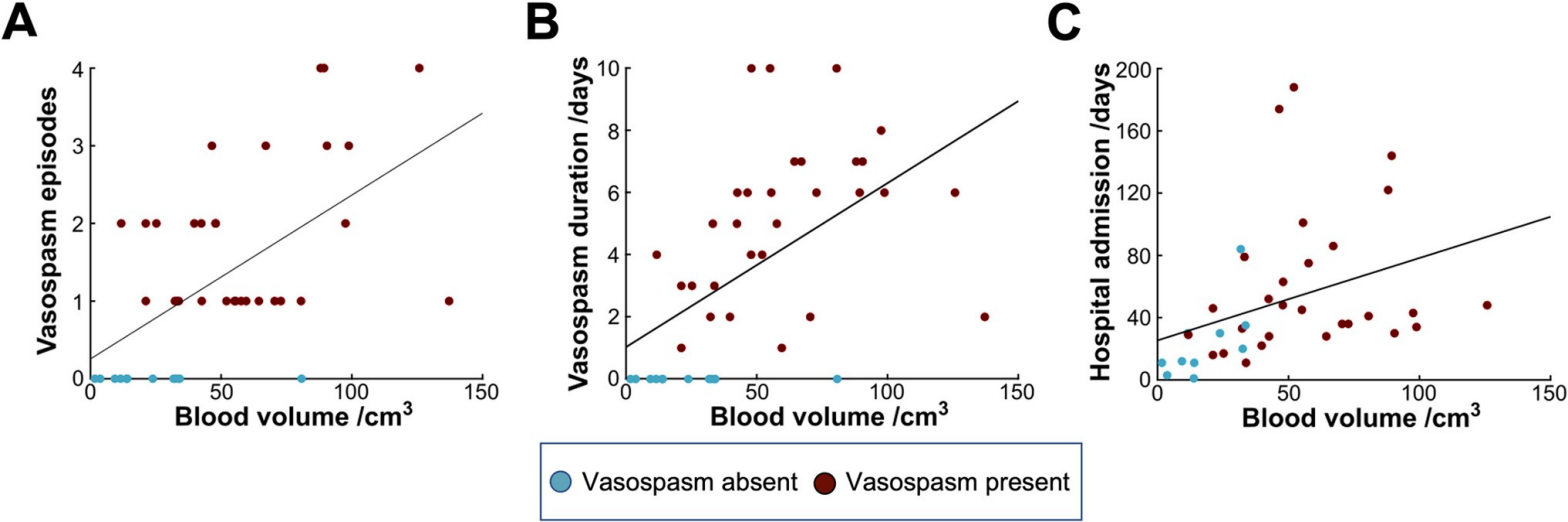
Associations between subarachnoid blood volume and temporal vasospasm-related outcomes. A: Scatter plot showing the number of discrete episodes of vasospasm against normalized blood volume. B: Scatter plot showing the duration of vasospasm in days plotted against normalized blood volume. C: Scatter plot showing the total length of hospital stay plotted against normalized blood volume.

Increased blood volume was significantly associated with a greater number of separate episodes of radiological vasospasm (r = 0.57, p < .001) [Fig 6A]. Similarly, there was a strong association between blood volume and vasospasm duration (r = 0.54, p < .001; Fig 6B). However, no association was found between subarachnoid blood volume and the time from admission date to first vasospasm (r = -0.13; p = 0.51).

### Association of subarachnoid blood volume with neurosurgical patient outcomes

Blood volume was not significantly associated with length of ITU stay (r = 0.16; p = 0.34), and there was no difference in mean blood volume between patients who subsequently died and those who did not, although this approached significance (fatality group: 77.9±43.9 cm^3^; non-fatality group: 47.0±30.2 cm^3^; t = 1.87; p = 0.07). However, blood volume was significantly correlated with total length of hospital stay (r = 0.36; p = 0.027; Fig 6C), indicating that patients with larger subarachnoid blood load may experience a more complicated or prolonged recovery.

## Discussion

### Summary

In this article, we present a novel ITK-SNAP-based pipeline for reliable and efficient segmentation of subarachnoid blood on initial CT head scans using semi-automated methods and expertly verified. Utilizing this framework, we show that greater total segmented blood volume following subarachnoid hemorrhage is significantly associated with a greater probability of subsequent radiological vasospasm, with the odds of vasospasm occurring increasing by approximately 7% for each cm^3^ of subarachnoid hemorrhage. We also demonstrate that subarachnoid blood load influences the natural history of vasospasm both in terms of duration and number of distinct episodes, and is associated with overall hospital length of stay.

### Interpretation and context

Our pipeline successfully produced segmentations of blood from CT head scans for all scans included in the study. Segmentations were close to ground truth as defined by corrected segmentations provided by a consultant neurovascular surgeon, indicating that our methods were accurately and reliably delineating blood from other tissue, in spite of its complex and tortuous morphology and also in spite of a wide array of scanner acquisition protocols from several referring hospitals. While previous machine learning methods have been used to detect and classify intracranial hemorrhages [37, 38], by providing saliency maps highlighting probable regions where blood is distributed [39], these methods do not produce segmentations from which precise blood volumes can be obtained. Quantitative volumetric segmentations have typically been applied to haemorrhagic lesions from traumatic brain injuries, with focus on subdural haematoma, extradural haematoma, and intraparenchymal hemorrhage [40–44]. Less work has attempted the automated segmentation of subarachnoid blood [38], and to our knowledge this work represents the first use of machine learning techniques to segment blood from CT head scans in aneurysmal SAH patients.

Our results demonstrate that the initial blood burden following subarachnoid hemorrhage has future consequences. Previous literature has focused on predicting the risk of symptomatic vasospasm (or delayed cerebral ischaemia) based on blood volume on CT head scans [4, 7, 8, 11, 12], with few papers addressing angiographic or radiological features. In our cohort, haemorrhagic blood load was associated with greater radiological vasospasm risk, episodes, duration, severity and a longer length of stay in hospital. Radiological vasospasm is itself well known to be strongly associated with delayed cerebral ischaemia and poorer functional outcomes [21, 22].

Interestingly, although we found a significant association between total blood volume and the radiologist’s impression of vasospasm severity, post-hoc testing with correction for multiple comparisons did not find a difference in blood volume between subgroups of patients with mild, moderate, and severe vasospasm. The relationship between blood load and vasospasm is likely complex, dependent on additional factors such as anatomical distribution of blood, and it may be that a larger sample size is required to detect associations between total blood load and subjective severity. Similarly, while we saw no signfiicant association between blood load and fatality, only four patients died in our cohort, making robust statistical inferences about this outcome challenging, and larger sample sizes are needed.

To address the clinical utility of our segmentations, we compared the predictive power of total subarachnoid blood volume with that of the widely-used modified Fisher scale. The modified Fisher scale alone possesses a number of limitations. Recent work has highlighted its inherent subjectivity, demonstrating only moderate inter-rater reliability scores [15]. Further, its predictive power is limited by its qualitative nature, resulting in a low-dimensional and low-resolution description of blood load and distribution. In our dataset, the modified Fisher scores also significantly predicted vasospasm risk but with reduced accuracy. In addition, we found that normalized blood volume provides additional information to the logistic regression model that is independent of the modified Fisher scale, and therefore may be incorporated into the future development of radiological vasospasm risk scales. Nonetheless, it may be that information about blood volume distribution across compartments (i.e. cisternal and ventricular blood) is provided by the modified Fisher scale, and this would not be captured by a total blood volume value. However, as blood segmentations can be further extended to include information about the spatial distribution of blood in the brain, we suggest that total blood volume provides a potential powerful regressor for predicting vasospasm.

### Limitations and strengths

Our interpretations are limited by the modest number of patients included, alongside the retrospective design of the study. Furthermore, the statistics presented here are exploratory, although the significance of the associations presented remained so after multiple comparison corrections. Additionally, we describe several steps to demonstrate that regression models fitted were robust, including the use of leave-one out cross validation methods. Although our fitted and internally validated regression model demonstrates good performance on our single-center dataset, further work with larger, multi-centre datasets will be required to cross-validate and confirm the findings reported above. Further, true blood volume may differ from that visible on standard CT images (for instance, thin blood layers outside the basal cisterns may be difficult to visualize and segment), further obscuring the notion of a ground truth blood volume for comparison. However, the results nonetheless highlight important and unexplored potential areas of further study within the vasospasm literature, and our presented analysis pipeline can easily be extended to larger datasets, prospective studies, collaborative segmentations, and more sophisticated statistical models.

The modified Fisher score was validated for prediction of delayed cerebral ischaemia [8] rather than radiological vasospasm, and so may not be expected to predict radiological vasospasm more accurately than blood volume. DCI remains challenging to diagnose, especially in sedated or high-grade patients [45], and unsurprisingly its onset is often missed [23]. Further, the sensitivity and specificity of TCD for detecting arterial vasospasm are variable and operator-dependent [22], and as such its findings cannot be considered ground truth. Rather, integrating information from a combination of different modalities—such as TCD, invasive neural monitoring and haemodynamic monitoring—can provide an effective pipeline for the prediction of poor outcomes such as DCI [46]. Indeed, early angiographic vasospasm is significantly associated with the subsequent development of DCI [47, 48], vasospasm as detected on TCD or CTA is well correlated with clinical deficits [21, 22, 47, 48], and both vasospasm duration and severity can be used alongside clinical assessment and course to assess the likelihood of delayed cerebral ischaemia and therefore the need for clinical interventions such as angioplasty [21, 24, 49, 50]. Therefore, we propose that one source of information for multimodal neuromonitoring could be objective imaging biomarkers such as total blood volume and distribution, alongside an estimated likelihood of radiological vasospasm, to guide clinical decision making and identify a subpopulation of patients that may require more stringent monitoring and/or intervention. Further modeling would be necessary to determine the influence of CSF drainage factors which may assist in reducing subarachnoid blood volume.

Fully manual segmentations drawn out by experts or trained raters, despite being considered the gold standard method, are time- and labor-intensive, often require lengthy training periods [51], and risk introducing substantial intra-rater and inter-rater variability and bias arising from various sources, including differences in opinion about the ground truth segmentation and in anatomical knowledge about relevant structures [52]. Accordingly, previous work has shown substantial variation in consistency and reproducibility of manual segmentations across both raters and structures [53–55]. Over recent years, semi-automated and automated methods have begun to match and even outperform manual segmentation in metrics of precision and inter-rater variability across a variety of modalities and structures [51, 54, 56, 57].

Our semi-automated method adds to the growing literature of potential applications for machine learning methods in radiological interpretation and triage, and removes some of this intra- and inter-rater variability. Many computer-assisted methods for delineation of blood volume have previously focused on segmentation of hemorrhages within non-subarachnoid space [40, 58–61]. However, application of these methods to aSAH has been noted to be challenging [41, 59], and accordingly Dice scores for segmentations of subarachnoid blood have been consistently lower than for other hemorrhage subtypes [38, 55, 62, 63], and convolutional networks used to automatically segment intracranial hemorrhage that includes subarachnoid blood have only achieved low-to-moderate Dice scores [64, 65].

We attempted to mitigate any bias on behalf of the rater through expert assessment and correction, and correspondingly our mean Dice score between original and corrected segmentation was high (0.92), indicating excellent agreement between rater segmentation and expert opinion that required minimal correction. This score was substantially larger than those comparing manual segmentations by different observers [55, 63], and in previous literature [38, 55, 62, 63]. In particular, Boers et al. [55] previously utilized similar methods to segment aSAH, but achieved only moderate Dice scores for its automated segmentations (mean Dice score 0.55, range 0.00–0.83). We note that a particular advantage of our pipeline was that it allowed for real-time quality control, as the segmenter can observe the active contour evolution evolve, likely contributing to our high Dice scores. Nonetheless, some variability remains, as labeling training data for the classifier and placement of seeds remain as manual steps that may lead to unintentional biases in volumes, and experts may display some variation in their manual segmentations. We anticipate that a greater number of experts, fully blinded to the patient condition, would provide more reliable and consistent segmentations to overcome the latter limitations.

Segmentations took on average 10–15 minutes per scan for the rater to perform. While manual segmentation workload was not explicitly compared in this study, this is notably faster than fully manual methods would be expected to take, while maintaining high agreement with our manual corrections. Further, manual segmentation speed depends on operator expertise and complexity of blood distribution; the same is not necessarily the case with semi-automated methods, and anecdotally we saw little variation in segmentation time as tortuosity of blood increased. However, 15 minutes remains a non-trivial period of time that may bottleneck scan reporting, and may therefore be unfeasible in clinical radiology contexts.

Potential extensions to a fully automated pipeline (such as nnU-Net [66], which has already seen use in brain tumor segmentation [67, 68]) would address a number of these limitations and allow for development of a clinically valuable toolkit. Validated, fully automated segmentations would allow for faster, reproducible and accurate delineation of blood distribution and volume, and may remove variability introduced in manual steps. Nonetheless, these methods still require training and validation, and this dataset serves as an important repository in facilitating this research.

## Conclusion

The semi-automated pipeline presented here robustly segments fresh blood from CT head scans on admission following subarachnoid hemorrhage. Our segmentation pipeline is significantly faster than manual segmentations, and demonstrates high accuracy when compared with expertly corrected volumes. Using these methods, we demonstrate that blood load following aSAH is associated with risk and timeline of radiological vasospasm. Notably, the odds of developing radiological vasospasm were greater for larger hemorrhage volumes, with a 7% increase in vasospasm odds per additional cm^3^ of blood on the scan, and observed vasospasm is likely to be more severe and persist for longer. Total blood volume constitutes an independent predictor for radiological vasospasm from the clinically employed modified Fisher scale, and carries potential for extension in the future to fully automated segmentation pipelines, and for the development of more sophisticated radiological risk scores for vasospasm.

## Supporting information

S1 File Supplementary material. Supplementary Methods and Supplementary Figures and Tables.
(DOCX)

## Abbreviations

AIC: Akaike information criterion
aSAH: aneurysmal subarachnoid hemorrhage
AUC: area-under-curve
BET: brain extraction tool
BIC: Bayesian information criterion
CT: computed tomography
CTA: computed tomography angiography
CTH: computed tomography (of head)
DCI: delayed clinical ischaemia
DICOM: digital imaging and communications in medicine
DSA: digital subtraction angiography
EHR: electronic healthcare record
EVD: external ventricular drain
HU: Hounsfield unit
ITU: intensive therapy unit
IVH: intraventricular hemorrhage
mFS: modified Fisher scale
PACS: picture archiving and communication system
ROC: receiver operating characteristic
TCD: transcranial Doppler
