# Supplementary material for: Predicting vasospasm risk using first presentation aneurysmal subarachnoid hemorrhage volume: A semi-automated CT image segmentation analysis using ITK-SNAP
Source: PLoS One. 2023 Jun 1;18(6):e0286485. doi: 10.1371/journal.pone.0286485 (PMC10234558; doi:10.1371/journal.pone.0286485)
Supplement: S1 File — Supplementary Methods and Supplementary Figures and Tables. (DOCX) [file pone.0286485.s001.docx]

Supplementary Material

#

# Supplementary Methods

## Data and statistical analysis

The Hosmer-Lemeshow test looks for significant differences in observed versus predicted outcomes of a model across its deciles of fitted risk values, whereas the Stukel test adds additional predictors derived from the fitted values of the model and tests for significance of these predictors, indicating poor fit. Both detect departures from linearity and unspecified interactions, and are well discussed elsewhere [[1,2](https://paperpile.com/c/EPzziz/iwoj+r9qH)].

# Supplementary Figures and Tables

**S1 Table: Logistic regression fit parameters using the modified Fisher score only.** Modified Fisher scores were dummy-coded before inclusion in the model, with mFS 1-2 grouped together as the referent. Note that estimates for logistic regression are given in the form of log odds.

|  | | **Estimate (log odds)** | **Confidence interval** | ***t* value** | ***p* value** |
| --- | --- | --- | --- | --- | --- |
| **Intercept** | | -0.40547 | [-1.6706, 0.8597] | 0.62814 | 0.53 |
| **Modified Fisher score** | **1-2** | Referent | | | |
|  | **3** | 1.0986 | [-3.1190, 2.3080] | 0.79354 | 0.43 |
|  | ***4**** | *1.9741* | *[0.3838, 3.5644]* | *2.433* | *0.015* |

42 observations, 39 error degrees of freedom. Dispersion: 1

Chi^2-statistic vs. constant model: 6.31, p-value = 0.0426

**S2 Table: Logistic regression fit parameters using the mFS scores and confounding variables.** Modified Fisher scores were dummy-coded before inclusion in the model, with mFS 1-2 grouped together as the referent. Note that estimates for logistic regression are given in the form of log odds.

|  | | **Estimate (log odds)** | **Confidence interval** | ***t* value** | ***p* value** |
| --- | --- | --- | --- | --- | --- |
| **Intercept** | | 2.8839 | [-2.3849, 8.1527] | 1.0728 | 0.28 |
| **Age** | | -0.08264 | [-0.1796, 0.0143] | -1.6702 | 0.09 |
| **Gender** | | 1.6176 | [-0.4870, 3.7221] | 1.5065 | 0.13 |
| **Treatment: coiled** | | -0.63353 | [-1.8616, 0.5945] | -1.0111 | 0.31 |
| ***EVD inserted**** | | *2.2294* | *[0.0721, 4.3867]* | *2.0255* | *0.04* |
| **Lumbar drain inserted** | | 0.12393 | [-2.011, 2.2490] | 0.11431 | 0.91 |
| **Modified Fisher score** | **1-2** | Referent | | | |
|  | **3** | -0.53881 | [-3.8684, 2.7908] | -0.31718 | 0.75 |
|  | **4** | 1.4268 | [-0.6977, 3.5513] | 1.3164 | 0.19 |

42 observations, 34 error degrees of freedom. Dispersion: 1

Chi^2-statistic vs. constant model: 14.7, p-value = 0.0399

AIC: 41.5505, BIC: 57.9723, AUC: 0.8583

Leave-one-out cross-validation: accuracy 69.1%, precision 75.8%, sensitivity 83.3%, F1 79.4%.

**S3 Table: Logistic regression fit parameters using the blood volume, mFS scores, and confounding variables.** Modified Fisher scores were dummy-coded before inclusion in the model, with mFS 1-2 grouped together as the referent. Note that estimates for logistic regression are given in the form of log odds.

|  | | **Estimate (log odds)** | **Confidence interval** | ***t* value** | ***p* value** |
| --- | --- | --- | --- | --- | --- |
| **Intercept** | | 3.7773 | [-3.3313, 10.8859] | 1.0415 | 0.30 |
| **Age** | | -0.11272 | [-0.2398, 0.0144] | -1.7378 | 0.08 |
| **Gender** | | 1.7815 | [-0.4559, 4.0188] | 1.5606 | 0.12 |
| **Treatment: coiled** | | -0.75851 | [-2.3043, 0.7873] | -0.96175 | 0.34 |
| **EVD inserted** | | 0.9748 | [-1.3261, 3.2757] | 0.83036 | 0.41 |
| **Lumbar drain inserted** | | 0.020753 | [-2.6246, 2.6661] | 0.015376 | 0.99 |
| ***Normalised blood volume**** | | *0.076197* | *[0.0008, 0.1516]* | *1.9798* | *0.048* |
| **Modified Fisher score** | **1-2** | Referent | | | |
|  | **3** | -3.0509 | [-7.7477, 1.6459] | -1.2732 | 0.20 |
|  | **4** | –0.27451 | [-2.9733, 2.4243] | -0.19936 | 0.84 |

42 observations, 33 error degrees of freedom. Dispersion: 1

Chi^2-statistic vs. constant model: 22.7, p-value = 0.00377

AIC: 41.5505, BIC: 53.7142, AUC: 0.9111

Leave-one-out cross-validation: accuracy 78.6%, precision 86.2%, sensitivity 83.3%, F1 84.8%.

# References

1. [Hosmer DW, Hosmer T, Le Cessie S, Lemeshow S. A comparison of goodness-of-fit tests for the logistic regression model. *Stat Med*. 1997;16(9):965-980.](http://paperpile.com/b/EPzziz/iwoj)

2. [Hosmer DW, Hjort NL. Goodness-of-fit processes for logistic regression: simulation results. *Stat Med*. 2002;21(18):2723-2738.](http://paperpile.com/b/EPzziz/r9qH)
